# Benchmark of Open‐Access Star‐Allele Callers to Accurately Assess Haplotypes and Phenotypes in Pharmacogenetic Studies

**DOI:** 10.1002/cpt.70365

**Published:** 2026-06-18

**Authors:** Marc B. Gros‐La‐Faige, Emmanuelle Génin, Anthony F. Herzig

**Affiliations:** ^1^ Univ Brest, Inserm, EFS, UMR 1078, GGB Brest France; ^2^ CHU Brest Brest France

## Abstract

Genetic polymorphisms are common in pharmacogenes, with sometimes important implications for drug metabolism. Assessing the correct enzyme phenotype from genetic data is thus a crucial step into the development of personalized medicine. Many bioinformatics star‐allele callers have been developed for this purpose of identifying the correct star alleles and the associated phenotype, each of them having their specific method and limitations. Despite the important benchmarks that have been made so far, their performances have not yet been fully explored depending on various parameters, such as the type of genetic data provided as input or the individuals' ancestry. Hence, we provide a multi‐gene, multi data‐type comparison of the accuracy of four commonly used and open‐access star‐allele callers: PyPGx, ursaPGx, PharmCAT, and Aldy. We found that PyPGx and Aldy are overall more performant than the others, except for *CYP2D6* where ursaPGx was the most accurate with its *CYP2D6* dedicated caller that relies on the Cyrius software. Comparing to the commercial solution DRAGEN, PyPGx, and Aldy showed better results, except for *CYP2D6* where DRAGEN performed best. When only SNP‐chip or low‐pass sequencing data is available, the use of imputation greatly improves the performance of star‐allele callers, allowing performance comparable to that achieved with sequencing data. We also analyzed how concordance between star‐allele callers varies depending on population ancestry. Our findings offer guidance on the choice of star‐allele caller, depending on the pharmacogene being studied and the resolution of available genetic data.

**Study Highlights**

**WHAT IS THE CURRENT KNOWLEDGE ON THE TOPIC?**

Many star‐allele callers have been developed to accurately identify star alleles in complex pharmacogenes, each of them often relying on different methods. Although several articles describe and compare their performance, they often limit their scope to the most complex pharmacogenes or to one type of genetic data.

**WHAT QUESTION DID THIS STUDY ADDRESS?**

This study evaluates the performances of some recent and widely used star‐allele callers in a more complete range of scenarios by comparing different types of genetic data from individuals of different ancestries for 10 genes.

**WHAT DOES THIS STUDY ADD TO OUR KNOWLEDGE?**

Statistical imputation, either from SNP array or low‐pass sequencing data, or SNP array data enriched in pharmacogenomic loci allows us to achieve performance similar to that of whole‐genome sequencing. Moreover, some star‐allele callers are more prone to discordances when individuals are of African ancestries.

**HOW MIGHT THIS CHANGE CLINICAL PHARMACOLOGY OR TRANSLATIONAL SCIENCE?**

These findings provide guidance for researchers and clinicians on which star‐allele caller to use depending on the pharmacogenes analyzed and the type of data they have access to. Developing and evaluating star‐allele callers should also be done on more diverse datasets to avoid an overrepresentation of European populations.

Pharmacogenetics is the study of genetic variations that influence individual response to medications.^1^ Being able to predict an individual's reaction to a drug based on their DNA sequence represents a major challenge in order to adapt each patient's care and avoid adverse drug reactions (ADR).^2^, ^3^ Hundreds of genes are known to harbor variants that can result in a variable drug response; these genes are referred to as pharmacogenes. Among them, 34 are currently annotated as “very important” by ClinPGx due to their pleiotropic effect in the metabolism of many drugs or their association with severe drug responses.^4^ The majority of these genes code for enzymes that act as catalysts in the liver, to either activate or eliminate the molecule introduced into the body by the medication.^1^ The metabolizer status or the functional activity of a given pharmacogene, usually depends on the combination of different variants grouped into functional haplotypes known as star (*) alleles.^5^, ^6^ For example, *CYP2D6* harbors more than a hundred of star alleles, defined by an unique combination of ~130 variants, each of them being associated with an activity score describing the functionality of the resulting enzyme.^7^ Accurate star‐allele calling is crucial to determine the pharmacogenetic profiles and adapt individual treatment.

To resolve the pharmacogenetic profile of an individual, many bioinformatics tools have been developed for calling star alleles in “very important” pharmacogenes (VIPs) using whole genome sequencing data or targeted microarrays, including freely available ones such as ursaPGx,^8^ PyPGx,^9^ Aldy,^10^, ^11^ and PharmCAT.^12^ All of these tools provide predicted diplotype combinations for various pharmacogenes, along with the corresponding phenotype predictions for certain tools (PyPGx and PharmCAT), and even related drug prescription guidelines in the case of PharmCAT (**Table** **1**). Although their primary objective is the same, to predict the diplotype combination of each individual, many factors can affect their accuracies, such as the method employed for star‐allele calling, the type of genetic data provided in input (microarrays or short‐read sequencing), the individual's ancestry; all of which have different levels of importance depending on the pharmacogene being analyzed. To date, comparisons of star‐allele calling tools have mainly focused either exclusively on *CYP2D6* ^13^, ^14^, ^15^ or by using Next‐Generation Sequencing (NGS) data.^10^, ^11^, ^16^, ^17^, ^18^, ^19^, ^20^, ^21^, ^22^, ^23^ Other works have also evaluated different type of data, such as Gan et al.^24^ who assessed the utility of Illumina global screening array chip, but without comparing different star‐allele callers, and Halman et al.^22^ who evaluated the impact of low depth sequencing on star‐allele callers' accuracy but did not consider statistical imputation. We propose an extension of those works by providing a multi‐tool, multi‐gene, multi data‐type comparison of star‐allele calling.

**Table 1:** Description of each star‐allele caller used in the study

Star‐allele caller	Requirements	Input data	Build	Number of genes	*CYP2D6* calling	SV detection	Phasing	Output
UrsaPGx	R	VCF	GRCh37	15	Done externally (Cyrius)	*CYP2D6* only	Need already phased VCFs	Diplotype prediction
		BAM (*CYP2D6* only)	GRCh38					
PharmCAT	Java & python	VCF	GRCh38	18	Done externally (StellarPGx or other)	*CYP2D6* only	Scoring system	Diplotype prediction
					Done internally (not recommended)	No		Phenotype prediction
								Guidelines
Aldy	Python	BAM	GRCh37	35	Done internally	Yes	Read‐based phasing	Diplotype prediction
			GRCh38					
PyPGx	Python	VCF	GRCh37	87	Done internally	Yes	Phasing using the external tool Beagle	Diplotype prediction
		BAM	GRCh38					Phenotype prediction

For our benchmark, we selected star‐allele callers that are freely available, capable of calling multiple genes using genotyping data, and are actively maintained at the time of writing. Based on these criteria, we included ursaPGx, PyPGx, and PharmCAT in our analysis. Although other tools satisfied the inclusion criteria, some were excluded for additional reasons. This is the case for stargazer, that was not included because it was developed by the same team that later proposed PyPGx, which uses similar ideas but with an improved methodology. In **Table** **S1**, we give more details on the tools selected or not and the criteria. We also added to the comparison a tool that processes WGS data; for this, we chose Aldy 4 as it was previously shown to be one of the most accurate among several WGS‐based tools across multiple genes.^10^, ^11^, ^16^, ^21^, ^22^ Callings were compared on the nine VIP genes supported by the four tools: *CYP2B6*, *CYP2C19*, *CYP2C9*, *CYP3A4*, *CYP3A5*, *CYP4F2*, *DPYD*, *NUDT15*, and *SLCO1B1* (**Table** **S2**) for 88 samples of the 1,000 genomes (1KGP) project^25^ provided by the Centers for Disease Control and Prevention (CDC)‐based Genetic Testing Reference Material Coordination Program (GeT‐RM).^26^ For each tool, we first compared the callings obtained for each individual against the star alleles provided by GeT‐RM. We also took advantage of the availability of DRAGEN calls on these same individuals to include in our comparison this commercial solution used in many labs. Then, we evaluated the impact of the type of data provided on star‐allele calling. We compared star‐allele calling accuracy when using as input (1) SNP array data from different sources, (2) imputed data from SNP array, and (3) imputed data from low‐pass WGS (0.5×) (LP‐WGS), or (4) WGS data (30×). Additionally, we studied the population‐specific concordance of all tools, as other studies showed that star‐allele calling accuracy varies depending on population ancestry.^27^ Finally, because *CYP2D6* is one of the most polymorphic PGx genes and is “estimated to contribute to the metabolism of ~ 20–25% of drugs,”^28^ callers adopt different solutions for *CYP2D6* identification. We thus analyzed this gene separately using the different *CYP2D6*‐specific methods proposed by each caller.

## MATERIALS AND METHODS

### Description of the different tools considered in the benchmark

#### ursaPGx (0.2.1)

ursaPGx is an R package that requires phased VCF files aligned to either the GRCh37 or GRCh38 reference genome. The program first determines the set of callable alleles based on the variants present in the data. For each haplotype, if a perfect match is found with the defining haplotype of a star allele, the corresponding star allele is assigned; otherwise, the haplotype is reported as ambiguous. Finally, ursaPGx outputs the phased diplotype for the studied gene on each sample. For *CYP2D6* calling, ursaPGx relies on an external tool, Cyrius,^14^ using BAM or CRAM files.

#### PyPGx (0.25.0)

PyPGx is a Python package that accepts VCF files aligned to GRCh37 or GRCh38, either phased or unphased. For unphased input, PyPGx performs statistical phasing using Beagle^29^ with haplotypes from the 1,000 Genomes projects (1KGP) as a reference. For genes whose star‐allele definitions include structural variants (SV), PyPGx incorporates an additional detection step using read depth and coverage information extracted from the BAM files and a machine learning approach trained on both real and simulated sequencing data from 1KGP and GeT‐RM. The tool then reports the diplotype assignment for each sample, along with the predicted phenotype when available.

#### Aldy (4.7)

Aldy is a Python program that takes as input SAM, BAM, CRAM, or VCF files mapped to either GRCh37 or GRCh38. From these files, the program jointly extracts small variants and estimates exon‐ and intron‐level copy number from normalized read depth profiles, enabling detection of both full‐gene and partial structural variants, including hybrid alleles. Variants and copy number profiles are then merged into haplotypes using a combinatorial optimization framework (Integer Linear Programming) that minimizes the distance between the hypothetical possible configurations and the observed sequencing data.^10^ The reconstructed haplotypes are matched to known star alleles, and Aldy reports the most likely diplotype assignment, including cases where multiple diplotypes are equally supported.

#### PharmCAT (2.15.4)

PharmCAT processes VCF files aligned to GRCh38 only. In a preprocessing step, the input VCF is filtered to retain only relevant positions and to normalize multiallelic and indel variants. Diplotype calling is then performed for each gene: if the data are phased, PharmCAT attempts to match each haplotype to defined star alleles, assigning a match score based on the number of concordant variants. Possible diplotypes are constructed, scored as the sum of the haplotype match scores, and the highest‐scoring diplotype(s) are reported. For unphased data, PharmCAT generates all possible genotype combinations before applying the same scoring procedure. The tool outputs the most likely diplotype(s) per gene for each sample, together with associated phenotype predictions where available, and provides drug prescription guidelines for a broad range of medications. By simply generating all possible phase combinations, PharmCAT does not take into account knowledge of haplotype frequencies. This is in contrast to PyPGx which employs the statistical phasing algorithm Beagle along with a haplotype reference panel. For *CYP2D6* calling, PharmCAT proposes two methods: (1) the “research mode” that takes a VCF file as direct input but is not recommended by the developers as it does not account for the many *CYP2D6* SVs; or (2) the use of an external caller, the one recommended in their documentation being StellarPGx.^30^

#### DRAGEN 4.4 PGx pipeline

A secondary analysis pipeline for calling star alleles offered by the commercial Illumina service DRAGEN. Precise details of the statistical method of the caller are yet to be published, nor is the code open source, though it can be inferred from broad details in an Illumina white paper^31^ that targeted alignment and alignment profile analyses are evoked to determine likely star alleles in WGS data.

### Data

The different tools were compared using WGS data from the 1,000 Genomes Project^25^ (1KGP) and PGx genotype data from the GeT‐RM project. The GeT‐RM project is an initiative led by the CDC with the main goal to provide genomic reference materials to improve the accuracy and reliability of genetic tests. Star‐allele diplotypes and phenotypes of 30 pharmacogenes are available for 137 individual samples,^26^ including 88 samples from 1KGP (https://www.cdc.gov/lab‐quality/php/get‐rm/reference‐materials.html), with up‐to‐date consensus using recharacterization for *CYP2C9*, *CYP2C19*, *CYP2D6*, *CYP3A4*, *CYP3A5*, *TPMT*, *NUDT15*, and other pharmacogenes from recent studies.^32^, ^33^, ^34^, ^35^, ^36^, ^37^ Given that some consensus were outdated due to the evolution of the star‐allele nomenclature, we replaced some consensus with a revised version provided by van der Maas et al.^38^ For our comparison, we only considered these 88 individuals for whom WGS data is also available. We focused the comparison on nine very important pharmacogenes (*CYP2B6*, *CYP2C19*, *CYP2C9*, *CYP3A4*, *CYP3A5*, *CYP4F2*, *DPYD*, *NUDT15*, *SLCO1B1*) that are called by all the four tools considered in the benchmark (**Table** **S2**). We analyzed *CYP2D6* separately, because PharmCAT and ursaPGx use different methods for this calling and can rely on external calling tools (StellarPGx and Cyrius, respectively). Depending on the gene, GeT‐RM data were available for either all the 88 1KGP individuals or only a subset of them (see first line of **Table** **2** that gives for each gene the number of individuals). Ambiguous diplotypes with more than two possible star alleles (such as “(*2;*10)/*6” for NA18945, *CYP2B6*) were discarded from the analysis. We also updated and standardized some star alleles in GeT‐RM: SLCO1B1*1B which became SLCO1B1*37, SLCO1B1*1A into SLCO1B1*1 and CYP3A4*1B into CYP3A4*1. Summing over the nine genes, a total of 682 diplotypes were available to serve as gold‐standard to benchmark the star allele calls obtained by the different tools. We gave already phased VCF and/or BAM files of WGS data from phase 3 1KGP as input to the different PGx tools. We used phased VCF because some star‐allele callers need phased data, and providing unphased VCF can significantly affects star‐allele calling accuracy. To benchmark the selected tools against a commercial solution that is used in many labs, we also downloaded DRAGEN 4.4^39^ calls on the 1KGP Phase 3 dataset at the following link: s3://1000genomes‐dragen‐v4‐4‐7/ (https://registry.opendata.aws/ilmn‐dragen‐1kgp/). We compared the diplotypes we obtained with the different tools and the DRAGEN calls to the GeT‐RM reference diplotypes.

**Table 2:** Concordance table with GeT‐RM per gene and per type of data. The number of no calls is highlighted in parentheses

Concordance with GeT‐RM											
Dataset	Star‐allele caller	CYP2B6 (*n* = 85)	CYP2C19 (*n* = 88)	CYP2C9 (*n* = 87)	CYP3A4 (*n* = 88)	CYP3A5 (*n* = 88)	CYP4F2 (*n* = 67)	DPYD (*n* = 88)	NUDT15 (*n* = 3)	SLCO1B1 (*n* = 88)	Overall
AMP tier 1/2	ursaPGx	0.0% (85)	87.5%	92.0%	90.9%	100.0%	59.7%	42.0%	66.7%	0.0% (88)	59.2% (173)
	PyPGx	0.0% (85)	88.6%	94.3%	90.9%	100.0%	59.7%	42.0%	100.0%	0.0% (88)	59.8% (173)
	PharmCAT	0.0% (85)	87.5%	73.6%	86.4%	100.0%	40.3%	42.0%	100.0%	0.0% (88)	54.5% (173)
GSA‐MD v3	ursaPGx	47.1%	0.0% (88)	93.1%	97.7%	100.0%	79.1%	56.8%	66.7%	81.8%	69.2% (88)
	PyPGx	47.1%	6.8%	93.1%	97.7%	100.0%	79.1%	70.5%	66.7%	81.8%	71.8%
	PharmCAT	43.5% (1)	0.0%	72.4%	93.2%	100.0%	56.7%	54.5%	0.0%	42.0% (1)	57.6% (2)
GSA v4 + ePGx	ursaPGx	48.2%	90.9%	96.6%	98.9%	100.0%	79.1%	55.7%	66.7%	80.7%	81.4%
	PyPGx	48.2%	92.0%	96.6%	98.9%	100.0%	79.1%	70.5%	100.0%	80.7%	83.6%
	PharmCAT	48.2% (1)	90.9%	89.7%	98.9%	100.0%	56.7%	54.5%	100.0%	42.0% (1)	73.3% (2)
Imputation (GSA‐MD v3)	ursaPGx	45.9%	89.8%	94.3%	98.9%	100.0%	88.1%	56.8%	66.7%	84.1%	82.1%
	PyPGx	45.9%	89.8%	96.6%	98.9%	100.0%	88.1%	70.5%	66.7%	84.1%	84.2%
	PharmCAT	45.9%	88.6%	89.7%	98.9%	100.0%	88.1%	54.5%	66.7%	43.2% (1)	75.8% (1)
Imputation (low‐pass)	ursaPGx	47.1%	89.8%	94.3%	98.9%	93.2%	89.6%	55.7%	66.7%	83.0%	81.2%
	PyPGx	47.1%	90.9%	94.3%	98.9%	93.2%	89.6%	69.3%	66.7%	83.0%	83.1%
	PharmCAT	47.1% (1)	89.8%	87.4%	98.9%	93.2%	89.6%	54.5% (1)	0.0%	43.2% (1)	74.8% (3)
WGS	ursaPGx	48.2%	90.9%	96.6%	100.0%	100.0%	89.6%	55.7%	66.7%	84.1%	83.0%
	PyPGx	48.2%	92.0%	98.9%	100.0%	100.0%	89.6%	70.5%	100.0%	84.1%	85.5%
	PharmCAT	48.2% (1)	90.9%	92.0%	100.0%	100.0%	89.6%	54.5%	100.0%	43.2% (1)	77.1% (2)
	Aldy	87.1%	92.0%	98.9%	100.0%	100.0%	65.7%	55.7%	100.0%	84.1%	86.1%
	DRAGEN	62.4%	92.0%	98.9%	100.0%	100.0%	71.6% (4)	59.1% (12)	100.0%	61.4%	81.1% (16)

To perform the same analysis but comparing the phenotypes, we assessed the phenotypes of GeT‐RM using diplotype to phenotype translation tables of the studied genes from ClinPGx (https://www.clinpgx.org/page/pgxGeneRef). Since no phenotype–diplotype translation table exists for *CYP3A4* and *CYP4F2*, these two genes were removed from this part of the study. Summing over the seven remaining genes, 527 phenotypes were available to serve as a gold standard. Considering that not all the PGx tools provide a phenotype prediction, we used the same translation tables to assess the predicted phenotypes obtained with ursaPGx and Aldy.

The WGS phased VCF of 1KGP used are freely available: (http://ftp.1000genomes.ebi.ac.uk/vol1/ftp/data_collections/1000G_2504_high_coverage/working/20201028_3202_phased/). The BAM files of 1KGP are also freely accessible: (http://ftp.sra.ebi.ac.uk/vol1/run). Even if the WGS VCF of 1KGP is very dense, some star‐allele defining SNPs were missing from the data, either because they are very rare or specific to a particular population, and thus not observed in 1KGP, or because they were of poor quality and so removed from the data by the 1KGP consortium.

To assess the impact of the type of data available on star‐allele calling, we simulated three other types of data in addition to the WGS data already available: (1) SNP‐chip data, (2) SNP‐chip genotyping data imputed with 1KGP and (3) simulated low‐pass data imputed with 1KGP. The SNP‐chip data (1) was simulated from the WGS phased VCF data by extracting positions present on the Illumina Global Screening Array (GSA) 24 V3.0 – Multi‐disease (MD) drop‐in panel (GSA‐MD v3) with 730,059 probes that we lifted over the GRCh38 using liftOver from the UCSC Genome Browser.^40^ We simulated in the same way two additional SNP‐chip dataset: one being the list of the Association for Molecular Pathology (AMP) tier 1/2 minimum sets of alleles for PGx testing, and the other one being the GSA 48 V4.0 with enhanced PGx (GSA v4 + ePGx), with a greater coverage of PGx gene for pharmacogenomic research. Starting from GSA‐MD v3 SNP‐chip data, we generated imputed data by using the 1KGP WGS phased data as a reference panel and imputing each sample individually in a leave‐one‐out manner: we first removed the sample and its relatives from the reference panel, to avoid imputing an individual with themselves or their family, and then performed the imputation. The imputation was done using Minimac4.^41^ Finally, we simulated low‐pass data imputed with 1KGP (3) by using samtools (1.16.1)^42^ view –s option to subsample BAM files of 1KGP to 0.5× depth. The seed used to subsample BAM files was 42. We then imputed each sample individually in a leave‐one‐out manner as described above. The imputation was done using GLIMPSE2 (v2.0.0).^43^, ^44^

Concordance for each tool was assessed by comparing the major star‐allele diplotype it reported with the reference diplotype. We assessed whether observed differences in concordance between tools or between type of input data were significant by using a McNemar test. Similarly, differences in concordance between populations were assessed using a permutation test.

See supplementary materials for detailed explanations.

## RESULTS

### Comparison of the output of the four tools against GeT‐RM reference data

**Figure 1:**
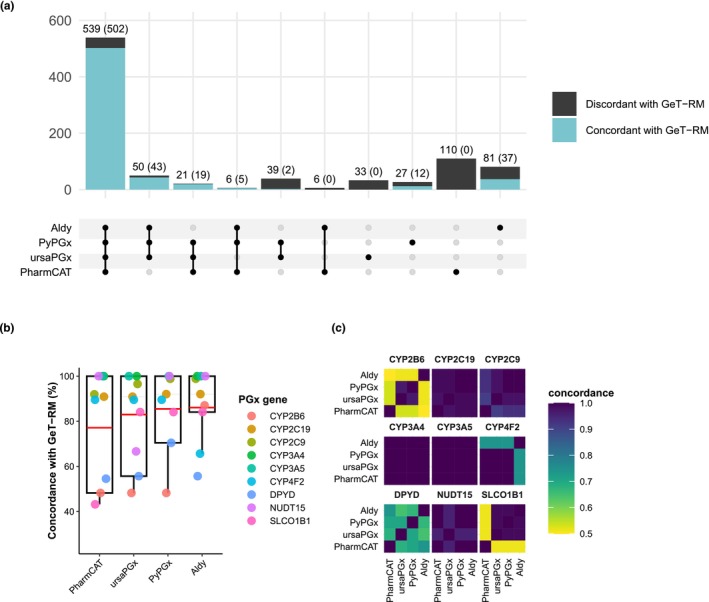
Accuracy of star‐allele callers with the GeT‐RM consensus for diplotypes. (**a**) Upset plot representing the intersections of star‐allele caller output for the 682 diplotypes. The lower part of the graph represents the intersection matrix, showing which intersection of tools is plotted in the above barplot that represents the size of the intersection. The exact total size of each intersection is printed above each bar. The number of calls in these intersections that are concordant with GeT‐RM are represented in blue, and printed in parentheses. (**b**) Diplotype concordance of each star‐allele caller with GeT‐RM consensus for each gene. The average diplotype concordance is represented in red. (**c**) Pairwise concordance of diplotype calls between each pipeline for each gene.

Diplotype and phenotype predictions obtained with each tool using the 1KGP sequencing data for the nine VIP genes were compared against the GeT‐RM data. The output of each pipeline along with the reference is provided in **Table** **S3**. The overall concordance between the star alleles diplotypes found by the different tools and GeT‐RM diplotypes is very high with a majority (73.6%, 502/682) of the 682 diplotypes correctly called by all four tools (**Figure** **1** **a**). For the prediction of phenotypes, the same was observed with a higher performance (83.7%, 441/527 of phenotypes correctly predicted by all four tools, **Figure** **S1**). Performances are better for the tools that can process BAM files (PyPGx and Aldy) compared to the tools that only process VCF files (ursaPGx and PharmCAT) (**Table** **S4**) (concordance across all genes of 86.1% (587/682), 85.5% (583/682), 83.0% (566/682), and 77.1% (526/682) for Aldy, PyPGx, ursaPGx, and PharmCAT, respectively). Moreover, only a minority of misidentified alleles have a high clinical impact. Indeed, only 9, 8, 13, and 18 of the misidentified star alleles by Aldy, PyPGx, ursaPGx, and PharmCAT, respectively, are in AMP tier 1/2 sets of minimal testing (**Figure** **S2**, **Table** **S5**). When compared to DRAGEN 4.4, PyPGx and Aldy were significantly more accurate, ursaPGx had a similar performance, and PharmCAT was significantly less accurate (**Figure** **S3**, **Table** **S4**).

When looking at the results per gene, we found that star‐allele calling accuracy is generally concordant between tools but there are some differences between genes (**Figure** **1** **b,c**). For three of the genes, *DPYD*, *CYP2B6*, and *SLCO1B1*, we observe more discrepancies between callers. For *CYP2B6*, the differences observed are explained by the fact that PharmCAT is unable to tell apart *6(rs3745274 + rs2279343), *9(rs3745274), *37(rs3745274 + rs2279343 + rs373489637), *38(rs3745274 + rs2279343 + rs281864907), *39(rs3745274 + rs2279343 + rs535039125), *40(rs3745274 + rs2279343 + rs200458614), *41(rs3745274 + rs2279343 + rs201500445), and *42(rs3745274 + rs2279343 + rs374099483) due to the lack of defining SNPs for those star alleles. In those cases, PyPGx and ursaPGx will report *9 only, as it is the only star allele fully matching the haplotypes, whereas Aldy will be able to tell apart *9 from *6 by identifying new SNPs from the read alignment files that are absent from the VCF, hence explaining why Aldy is more accurate for *CYP2B6* than the other tools. *DPYD* is a highly polymorphic gene, with many SNPs affecting the enzyme metabolism. Discrepancies observed in *DPYD* calling are driven by different factors. First, the tools target different variant sets for *DPYD* (list of *DPYD* variants identified by each star‐allele caller in **Table** **S6**), that could lead to miscalls. Another source of discrepancies is the type of input data: while Aldy have access to every SNPs present in the alignment files, the other tools will not have access to SNPs filtered out after quality control. Finally, the greatest source of discordance for *DPYD* is the lack of formal star‐allele nomenclature. If more than one SNP are present on the same haplotype, Aldy and PharmCAT will report every SNPs of the haplotype (e.g., for NA10831, Aldy's and PharmCAT's calls are rs1801159 + rs17376848/rs1801265, respectively), while PyPGx will report only the SNP with the greatest clinical impact (rs1801159/rs1801265 for NA10831) and ursaPGx will call the haplotype ambiguous because it does not match any of the known *DPYD* haplotypes (*Amb/rs1801265 for NA10831). This problem could be easily avoided by the creation of a clear star‐allele nomenclature for *DPYD* for a better standardization of more complex *DPYD* haplotypes (such as rs1801159 + rs17376848), instead of letting each star‐allele caller decide what to report in ambiguous cases like this. Moreover, the reference dataset for *DPYD* includes only a small set of variants, and almost never reports haplotypes with more than one alternative SNP. This lead to misleading poor performances of star‐allele callers for this gene when compared to GeT‐RM. For *SLCO1B1*, PharmCAT also has difficulties calling this gene, especially because it is unable to differentiate *37(rs2306283) and *42(rs2306283 + rs141467543). In the absence of rs141467543 information in the VCF file, as it is the case here (probably because it is a very rare variant, AF = 7.027 × 10^−5^ in GnomAD v4.1.0^45^), PharmCAT attributes *37 and *42 the same score, and reports both as it cannot tell them apart, whereas the other tools only report *37 because it is the only matching star allele when only rs2306283 is present.

### Impact of the type of input data on diplotype calling accuracy

**Figure 2:**
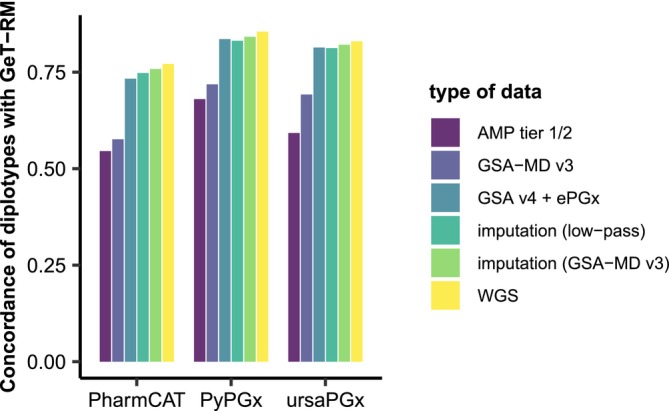
Concordance of star‐allele callers between different data types with GeT‐RM for diplotype and for each pipeline.

To measure the impact of the type of data provided on star‐allele calling, we compared the diplotype concordance of the output provided by PyPGx, ursaPGx, and PharmCAT using (1) SNP‐chip genotyping data from GSA‐MD v3, (2) GSA v4 + ePGx, (3) AMP tier 1/2 coverage, (4) SNP‐chip genotyping data (GSA‐MD v3) imputed with 1KGP, (5) simulated low‐pass data imputed with 1KGP, and (6) WGS data (VCF only) and compared the results against the GeT‐RM gold‐standard data. Aldy was excluded from this section because it was designed for NGS data (Aldy needs a sequencing profile as input [a copy‐number profile, either already implemented in Aldy, or generated with Aldy from an alignment file, see https://github.com/0xTCG/aldy], which cannot be provided when using simulated microarray data and because Aldy's developers recommend using their software with SAM/BAM files for best results (https://github.com/0xTCG/aldy)). Concordance with GeT‐RM is significantly lower when only genotyping data from GSA‐MD v3 or from AMP tier 1/2 coverage is available (**Figure** **2**, **Table** **S7**). This is true for all of the three tools. The low concordance when using AMP tier 1/2 coverage comes mainly from *CYP2B6* and *SLCO1B1* (concordance with GeT‐RM at 0% for all tools) because no alleles of these two genes are present in the sets of minimum testing proposed by AMP, despite their major influence on response to antidepressants and statins, respectively. To a lesser extent, *CYP4F2*, *DPYD*, and *CYP3A4* also had a drop in accuracy ranging from 10% to 30% discordance with WGS, due to the limited number of star alleles recommended for testing. The differences observed between GSA‐MD v3 and GSA v4 + ePGx come mainly from *CYP2C19*, for which star‐allele callers performed very poorly with GSA‐MD v3 (0%, 6.8%, and 0% concordance for ursaPGx, PyPGx, and PharmCAT, respectively). This is due to the lack of rs3758581 in GSA‐MD v3, a frequent SNP (93.87% in gnomAD) present in almost all star‐allele definitions of the gene. Interestingly, SNP chip genotyping data enhanced with PGx loci (GSA v4 + ePGx), or imputed data either from GSA‐MD v3 or from LP‐WGS give results that are very close to what is achieved with WGS compared with genotyping data, with only a minor significant drop in accuracy. Imputation from SNP‐chip and imputation from low‐pass sequencing data always provide similar results that are not significantly different, and using GSA v4 + ePGx data provides results as good as imputation either from GSA‐MD v3 or from LP‐WGS (**Figure** **2**, **Table** **S7**).

### Study of the tool concordances depending on the population

**Figure 3:**
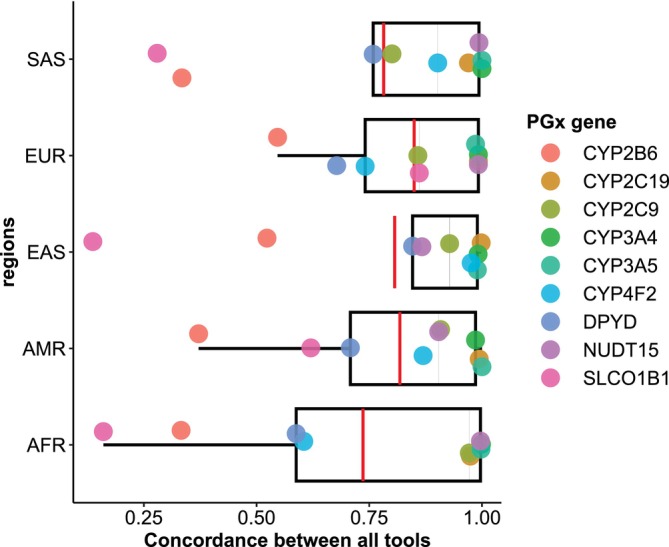
Boxplot of concordance of star‐allele callers between themselves per geographic regions using WGS data of 1KGP project. Each dot represents one gene. The average concordance per region is represented by a red line.

As the frequency of star alleles is highly dependent on the population, with some star alleles being population specific, we wanted to compare star‐allele callers' performances depending on the population studied. Given that the 88 reference samples from GeT‐RM represent a very small dataset to observe population differences, we extended our study to all the 1KGP samples (3,202 individuals) and compared how the tools agree depending on the population of origin of the individuals (**Figure** **3**). Here, we gave each tool phased VCF and/or BAM files of the 1KGP project. For a given diplotype, we calculated the concordance across all tools: 1 if all the tools gave the same result, and 0 otherwise. We then averaged these concordance statistics across all diplotypes for each population and for each pharmacogene. We find that the tools are least concordant in the African‐ancestry individuals (AFR) with 73.6% concordance on average against 85.0% for the European‐ancestry individuals (EUR) where the tools are the most concordant. By using a permutation test (*n* = 5,000), we observe that this difference in concordance is highly significant (*P*‐value <1/5,000 with none of the permutations showing a difference in concordance higher than the one observed) (**Figure** **S4**). The decreased concordance across tools in the AFR is driven by *SLCO1B1* and the previously described ambiguity between *37 and *42 for PharmCAT. The defining SNP of *37, rs2306283, is more frequent in AFR than in EUR (77.41% vs. 37.28% in gnomAD) leading to more ambiguity in AFR. The same problem arises for *CYP2B6*, where PharmCAT cannot tell apart *6, *9, *37, *38, *39, *40, *41, and *42 due to the lack of defining SNPs. The common defining SNP of all those star alleles, rs3745274, is also more frequent in AFR than in EUR (37.71% vs. 24.08%, from gnomAD). Some discordances are also explained by Aldy's ability to call new star alleles from non‐previously defined haplotypes.

In addition to *CYP2B6* and *SLCO1B1*, *DPYD* also exhibits an intermediate level of consensus between star‐allele callers, ranging from 84.6% of consensus calls in EAS to 58.8% in AFR. Discrepancies observed in *DPYD* calling are driven by different factors, such as the different sets of targeted variants and the lack of star‐allele nomenclature for this gene. Even though the origin of miscalls for this gene differ from *CYP2B6* and *SLCO1B1*, the lack of consensus observed still highlight the progress that can be made to improve *DPYD* characterization and downstream recommendations.

The concordance of the four tools was always significantly different between any pairs of regions, except between EAS and AMR which have both very similar concordances (80.6% and 81.8%, respectively) (**Table** **S8**).

### Performance of *CYP2D6* calling of the four tools against GeT‐RM reference data

**Figure 4:**
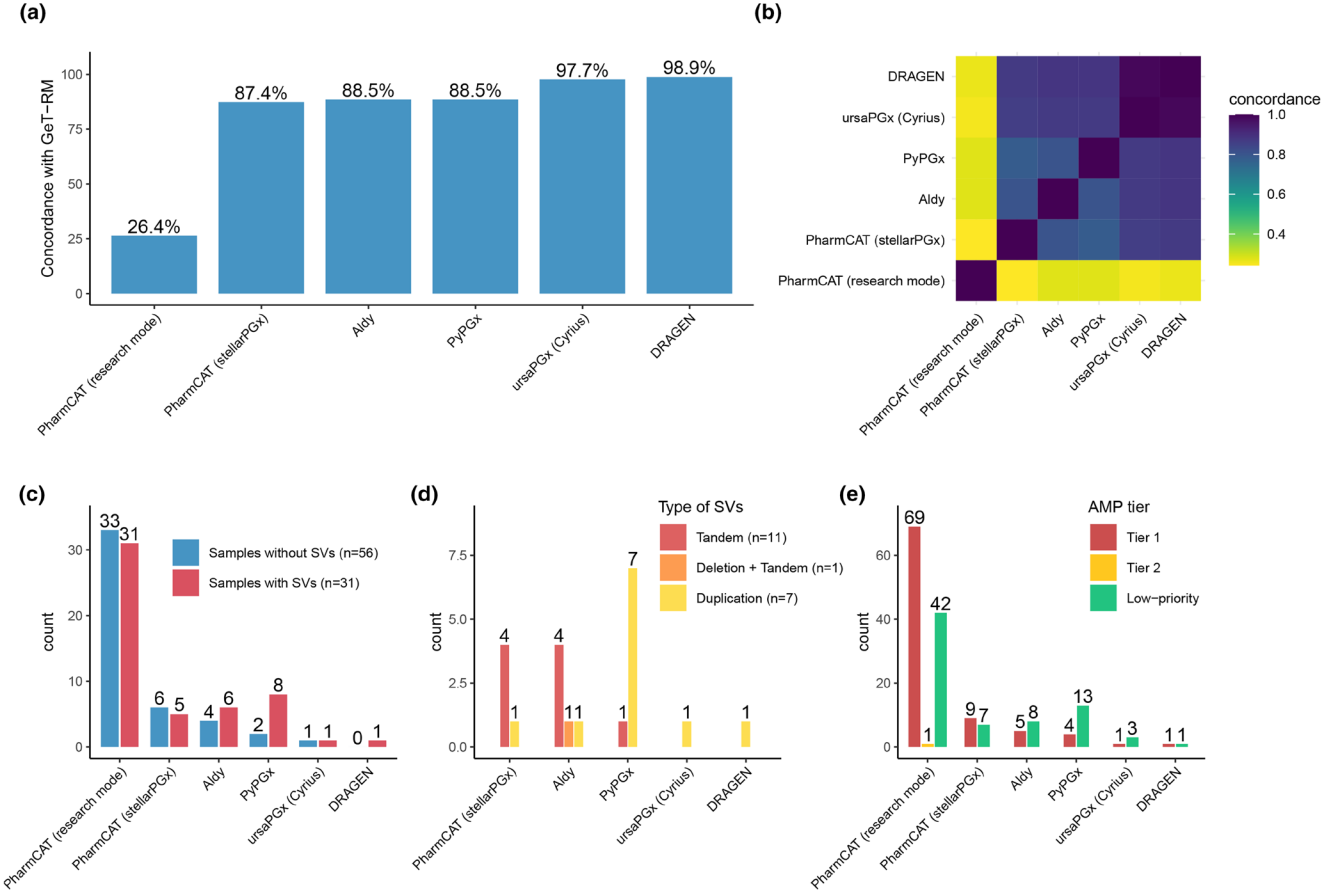
Accuracy of star‐allele callers with the GeT‐RM consensus for *CYP2D6* diplotypes. (**a**) Barplot showing the accuracy (concordance of diplotypes with GeT‐RM) of each star‐allele caller considered as well as calls from DRAGEN for *CYP2D6* calling. (**b**) Pairwise diplotype concordance matrix between each star‐allele caller for *CYP2D6*. (**c**) Samples with discordant diplotypes. The red color depicts samples with SVs in the reference diplotype, while the blue color represents cases without SVs in the reference diplotype. (**d**) Discordant diplotypes by SV type. The red color represents SVs considered as tandem, the orange color represents SVs considered as deletion + tandem, and the yellow color represents SVs considered as duplications. (**e**) Discordant star‐alleles stratified by AMP relevance. The red and yellow colors represent star‐alleles classified as AMP tier 1 and AMP tier 2, respectively. The green color represents misidentified star alleles that are not present in AMP sets of minimum testing.

*CYP2D6* is one of the most polymorphic pharmacogenes, with many different SVs affecting its function: full deletions or duplications (*5, *1×2), tandem SVs involving hybrid recombination with the paralog gene *CYP2D7* (*68 + *4, *36 + *10, *13 + *1), or both of the above at the same time (*5/*68 + *4, *1×2/*68 + *4). Because of their presence, many different strategies are adopted by star‐allele callers to call *CYP2D6* diplotypes. We thus explored the different solutions proposed by each star‐allele callers. All the individual calls from all star‐allele callers are available in **Table** **S9**. We observe that PharmCAT in “research mode” performs very poorly compared to all the other callers. PharmCAT (StellarPGx), Aldy, and PyPGx performed very similarly, with an accuracy of 87.4%, 88.5%, and 88.5%, respectively. The most accurate of all four tools is ursaPGx with its specific Cyrius caller that achieved an accuracy of 97.7% (**Figure** **4** **a**), similar to the one obtained with DRAGEN (98.9%) (**Figure** **4** **a**, **Table** **S10**). Even though PharmCAT (StellarPGx), Aldy, and PyPGx have almost the same concordance with GeT‐RM, they are not discordant for the same samples (**Figure** **4** **b**). When looking at the discordant cases, we observe that approximately half of the discordant samples for every caller harbor a diplotype with SVs, and even more than half for Aldy and PyPGx (**Figure** **4** **c**). PharmCAT (research mode) misidentified all samples with SVs, which was expected given that no SVs could be detected through VCF files only. When looking at the subset of misidentified samples harboring SVs, we observe differences in the type of SVs star‐allele callers struggle with (**Figure** **4** **d**). While PharmCAT (StellarPGx) and Aldy misidentified more tandem SVs, PyPGx was less accurate with whole duplications (**Figure** **4** **d**). Finally, when looking at the clinical relevancy of misidentified star alleles, we find that, whatever the mode, PharmCAT performs poorly on star alleles classified AMP tier 1, (with 69 and 9 misclassified alleles for the “research mode” and StellarPGx, respectively). Aldy and PyPGx were more accurate on AMP tier 1 star alleles (5 and 4 misidentified alleles, respectively), while, in comparison, ursaPGx (Cyrius) only misidentified one AMP tier 1 allele (**Figure** **4** **e**).

## DISCUSSION

Accurately calling star alleles is an essential step before providing recommendations for the physician to make informed and personalized prescriptions. However, star‐allele calling depends on many parameters, and pharmacogenetic clinical implementation is facing a cost–benefit dilemma. Consequently, we have benchmarked ursaPGx, PyPGx, Aldy, and PharmCAT to see their performances under different conditions.

We first assessed the accuracy of each algorithm by comparing their output to a reference panel provided by the GeT‐RM project. Overall, the pipelines using alignment files (BAM) in the star‐allele calling process are the most accurate, with Aldy being the most accurate in diplotype calling (85.6%) and PyPGx being the most accurate in phenotype prediction (98.7%). Even if Aldy and PyPGx are able to detect and call SVs, their better performances are not explained by the presence of SVs as no SVs are present in the 88 GeT‐RM individuals for the pharmacogenes analyzed. The differences are rather explained by the methods employed by each: PharmCAT scoring system is prone to call multiple diplotypes and thus to make errors, while Aldy, by looking at the BAM files directly, can detect variants that may have been filtered out by QC in the VCF and thus can call more accurately many star alleles in addition to star alleles with definitions that involve structural variation. The differences observed between pipelines for *SLCO1B1*, *CYP2B6*, and *DPYD* highlight the limitations of some methods. For example, the scoring system of PharmCAT may not be the most suited for star‐allele calling for some genes, even when using WGS data. The discordances in *DPYD* also highlight the difficulties of calling this gene, especially due to the lack of star‐allele nomenclature, leading each tool to have its own method of reporting *DPYD* variants. Comparing our results with previous benchmarks, we observed similarities, but also discordances. The per‐gene concordances of Aldy when using WGS data were very similar to the one observed by Halman et al.,^22^ with small differences that could be explained by the differences in the reference dataset used. Whereas the results obtained by Shugg et al.^21^ when using Aldy 4 with WGS are better than ours (concordance of diplotype calls of 99.7% across 14 genes). This can be due to the use of a different reference dataset, made of patients from the same clinic, and thus likely with less diversity than the 1KGP reference dataset we used, wherein we observed significant differences in accuracy between ancestry groups. We also found a lower accuracy of PharmCAT than Gan et al.^24^ when using the illumina GSA array (diplotype concordance ranging from 87.5% to 100%). This can be explained by the fact that Gan et al. used a “combination of PharmCAT and in‐house custom script to resolve ambiguous genotypes,” which is the main source of discordances for PharmCAT. The evolution of technological approaches in DNA sequencing and targeted genotyping have led to the emergence of various strategies to generate genetic information. Their differing characteristics lead to the requirement of different approaches for PGx studies and carry inherent strengths and weaknesses. Some studies have already illustrated that imputation could be a reliable method to infer the drug metabolism activity of a given pharmacogene,^46^, ^47^ but were focused on the imputation quality at the SNP level rather than the star allele or the diplotype levels. The same is true for low‐pass sequencing, where Wasik et al.^48^ concluded that low‐pass sequencing is a “competitive alternative to genotyping arrays,” but the authors were again looking at the SNP level only and not at the downstream star alleles. Hence, we compared the performances of each star‐allele caller depending on the type of data provided. We compared genotyping data from AMP tier 1/2 coverage, GSA‐MD v3, and GSA v4 + ePGx, imputation from LP‐WGS and from GSA‐MD v3, and WGS (30×) to the GeT‐RM reference panel. Interestingly, GSA v4 + ePGx, or imputation from either LP‐WGS data or GSA‐MD v3, were found to achieve performances that were almost as good as the ones obtained from WGS data. This result suggests that those methods could be a good trade‐off between cost and accuracy. Halman et al.^22^ assessed the impact of sequencing depth on star‐allele calling accuracy of Aldy, StellarPGx, Stargazer, and Cyrius, and concluded that performances decreased particularly at 5× depth. However, they used the raw LP‐WGS data and did not perform any imputation as we did. We agree with them that LP‐WGS would result in poor star‐allele callings but we showed that LP‐WGS combined with imputation provides reliable results and could be an interesting alternative to limit costs.

In addition to the nine VIP genes included in our analysis, we also tested the performances of the different tools for *CYP2D6*. Because of its high level of polymorphism, *CYP2D6* star‐allele calling is challenging and several star‐allele callers have been specifically designed for *CYP2D6* calling. Many previous works assessed star‐allele callers' accuracy on *CYP2D6*, based on NGS data^11^, ^13^, ^14^, ^15^, ^22^, ^30^ including comparisons to the reference dataset GeT‐RM. Across all studies, Aldy, StellarPGx, and Cyrius consistently achieved more than 80% concordance for *CYP2D6* when applied to WGS data, which is coherent with our results. Discrepancies regarding which tool performs best vary between studies and can be attributed to differences in the reference dataset used and to the specific software versions evaluated. We found that ursaPGx (Cyrius) was the most accurate, just behind DRAGEN. Interestingly, we found that PharmCAT (StellarPGx), Aldy, and PyPGx all have difficulties for SV identification but not for the same types of SV, suggesting that those callers could be improved or used in a complementary manner.

Even if developers of star‐allele callers have made efforts to make their tools user‐friendly, they still require a level of bioinformatics expertise that is not available in all laboratories. This may explain the popularity of commercial solutions such as the one proposed by Illumina with its DRAGEN secondary analysis suite, which enables PGx calling. To benchmark the results, we obtained with our selected callers against DRAGEN, we took advantage of the availability of DRAGEN 4.4 PGx calling results on 1KGP individuals to see how they compared to the GeT‐RM reference data. We found that DRAGEN achieved a low concordance with GeT‐RM compared to PyPGx and Aldy for all genes except for *CYP2D6*, where DRAGEN was the most accurate.

Given that pharmacogenomics profiles are highly variable between ancestries, we assessed star‐allele caller concordance between the different 1KGP populations. Pipelines were the most concordant in EUR (85.0%) and the least concordant in AFR (73.6%). Even though it is expected that Africans populations have the lowest concordance among tools given their higher level of pharmacogenomic diversity, it is still interesting to understand where those differences come from in order to improve star‐allele calling accuracy in those populations. These discordances are mainly driven by PharmCAT, which was prone to make errors in AFR, and by Aldy which can call new haplotypes without prior knowledge of their biological relevance. This ability can be useful for identifying new haplotypes and star alleles. However, its value is limited by the inability to predict the phenotype associated with these new haplotypes. This problem raises the question of pharmacogenetic accessibility to every ancestry, and their representations in actual pharmacogenomics studies.

One limitation of our study is the panel GeT‐RM used as a gold standard. A far larger reference panel, with more populations represented, would be greatly beneficial for future pharmacogenomics studies. Moreover, all the PGx tools described in this study have been developed using the GeT‐RM panel as a gold standard. PyPGx machine learning for SV detection was trained on this same dataset. Aldy was also used to characterize GeT‐RM reference materials for the 137 DNA samples for *CYP2C8*, *CYP2C9*, and *CYP2C19*.^34^ Moreover, the recharacterized calls from van der Maas et al.^38^ were also assessed using Aldy and PyPGx in addition to other tools. This may lead to a bias toward Aldy and PyPGx having better accuracy. However, additional analysis to check for this possibility using only the GeT‐RM consensus without this new recharacterization led to similar results, suggesting that the high accuracy of Aldy and PyPGx is not due to these recharacterizations (**Table** **S11**). Additionally, it could be possible to create an improved reference panel by obtaining long‐read sequencing (LRS) data for accessible samples and establishing a consensus calling from tools that can process and take full advantage of LRS data (Aldy, PyPGx, StarPhase,^49^ TAS‐LRS^18^) based on the same approach as the GeT‐RM reference used in this study. This was however outside the scope of this work.

Another limitation is the number of VIP genes missing from our study because we focused our study only on genes that were called by all considered tools, especially complex genes such as *UGT1A1* for which the highly polymorphic INDEL rs3064744, present in most star‐allele definitions of the gene, can be hard to accurately call or identify, especially due to INDEL normalization. Moreover, for some of the PGx genes included in our study, we were able to analyze only a very limited number of samples. This was the case for *NUDT15* for which we could only analyze three samples because in the GeT‐RM reference dataset only four samples out of the 137 had *NUDT15* diplotypes and among these four samples only three were in the 1KGP dataset. These three samples harbor either *3(rs116855232) or *6(rs746071566) for *NUDT15*. In the absence of information on the genotype at rs746071566, PharmCAT cannot identify *6 nor tell apart *3 from *2(rs116855232 + rs746071566), explaining its 0% concordance.

Finally, we only tested Aldy 4 as a fully WGS‐based star‐allele caller and did not consider other ones such as StellarPGx. Previous works from Hari et al.^11^ and Halman et al.^22^ showed that Aldy performs as accurately as StellarPGx and other star‐allele callers for many genes, and we thus chose Aldy as it can call more pharmacogenes.

## CONCLUSION

In this study, we compared the performances of four frequently used pharmacogenetic star‐allele callers for nine VIP pharmacogenes known to interact with a wide range of drugs, depending on the population studied and the type of data provided. Our results showed that Aldy and PyPGx were in general better than ursaPGx and PharmCAT. While the commercial caller DRAGEN can represent a good solution for a simple and accurate calling of many pharmacogenes, we showed that Aldy and PyPGx had better performances than DRAGEN, with the exception however of *CYP2D6* where DRAGEN was the most accurate. Furthermore, star‐allele calling accuracy was variable across genes, showing that there is still room for improvement, especially for *CYP2B6* and *DPYD*. Whereas WGS data is better for PGx studies, it is possible to achieve a similar level of precision using SNP chip designed for PGx studies or enriched through statistical imputation. Finally, calling *CYP2D6* remains a challenge that can be essentially addressed when alignment files are available, with ursaPGx (Cyrius) being the most accurate solution of our study.

## FUNDING

No funding was received for this work.

## CONFLICT OF INTEREST

The authors declared no competing interests for this work.

## AUTHOR CONTRIBUTIONS

M.B.G‐L‐F., E.G., and A.F.H. wrote the manuscript. M.B.G‐L‐F., E.G., and A.F.H. designed the research. M.B.G‐L‐F. performed the research. M.B.G‐L‐F. analyzed the data. All authors have read and approved the final manuscript.

## Supporting information


Figure S1.

Table S1.

## Data Availability

The genetic data used in this study can be found at http://ftp.1000genomes.ebi.ac.uk/vol1/ftp/data_collections/1000G_2504_high_coverage/working/20201028_3202_phased/ and http://ftp.sra.ebi.ac.uk/vol1/run/. The curated table from GeT‐RM diplotypes is available in **Table** **S3**. The raw GeT‐RM data used in this study can be found at https://www.cdc.gov/lab‐quality/php/get‐rm/reference‐materials.html. The 1,000 Genomes Phase 3 Reanalysis with DRAGEN 4.4 was accessed from https://registry.opendata.aws/ilmn‐dragen‐1kgp. The AMP tier 1/2 sets of minimum testing were downloaded from https://www.clinpgx.org/ampAllelesToTest. The code for generating the genotyping data, imputation data, and imputation with low‐pass data, and for running each pipeline is available at https://github.com/marcglf/benchmark‐star‐allele‐callers.
