# Supplementary material for: Benchmark of Open‐Access Star‐Allele Callers to Accurately Assess Haplotypes and Phenotypes in Pharmacogenetic Studies
Source: Clin Pharmacol Ther. 2026 Jun 18;120(2):520–30. doi: 10.1002/cpt.70365 (PMC13339515; doi:10.1002/cpt.70365)
Supplement: Supplementary file 1 — Figure S1. [file CPT-120-520-s002.docx]

**Supplementary Material**

**Benchmark of open-access star-allele callers to accurately assess haplotypes and phenotypes in pharmacogenetic studies**

Marc B. Gros-La-Faige^1^, Emmanuelle Génin^1,2,*^, Anthony F. Herzig^1,*^

^1^Univ Brest, Inserm, EFS, UMR 1078, GGB, F-29200 Brest, France

^2^CHU Brest, Brest, France

^*^These authors contributed equally to this work

**Figure S1.** Accuracy of star-allele callers with the GeT-RM consensus for phenotypes.

**
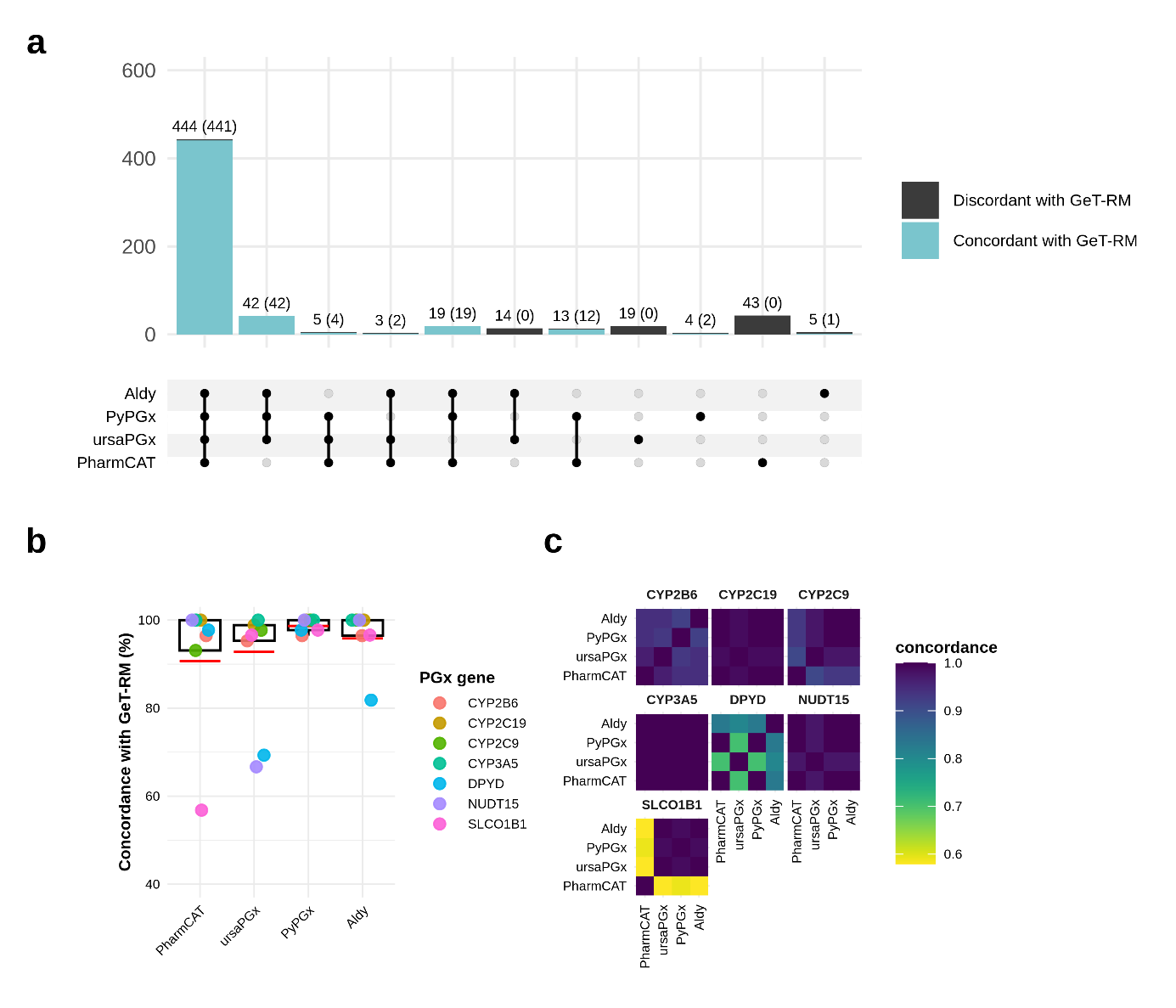
**

**a**: Upset plot representing the intersections of star-allele caller output for the 527 phenotypes. The lower part of the graph represents the intersection matrix, showing which intersection of tools is plotted in the above barplot that represents the size of the intersection. The exact total size of each intersection is printed above each bar. The numbers of calls in these intersections that are concordant with GeT-RM are represented in blue, and printed in parenthesis. **b**: Phenotype concordance of each star-allele caller with GeT-RM consensus for each gene. The average phenotype concordance is represented in red. **c**: Pairwise concordance of phenotype calls between each pipeline for each gene.

**Figure S2.** Clinical relevancy of misidentified star-alleles across the 9 genes considered.


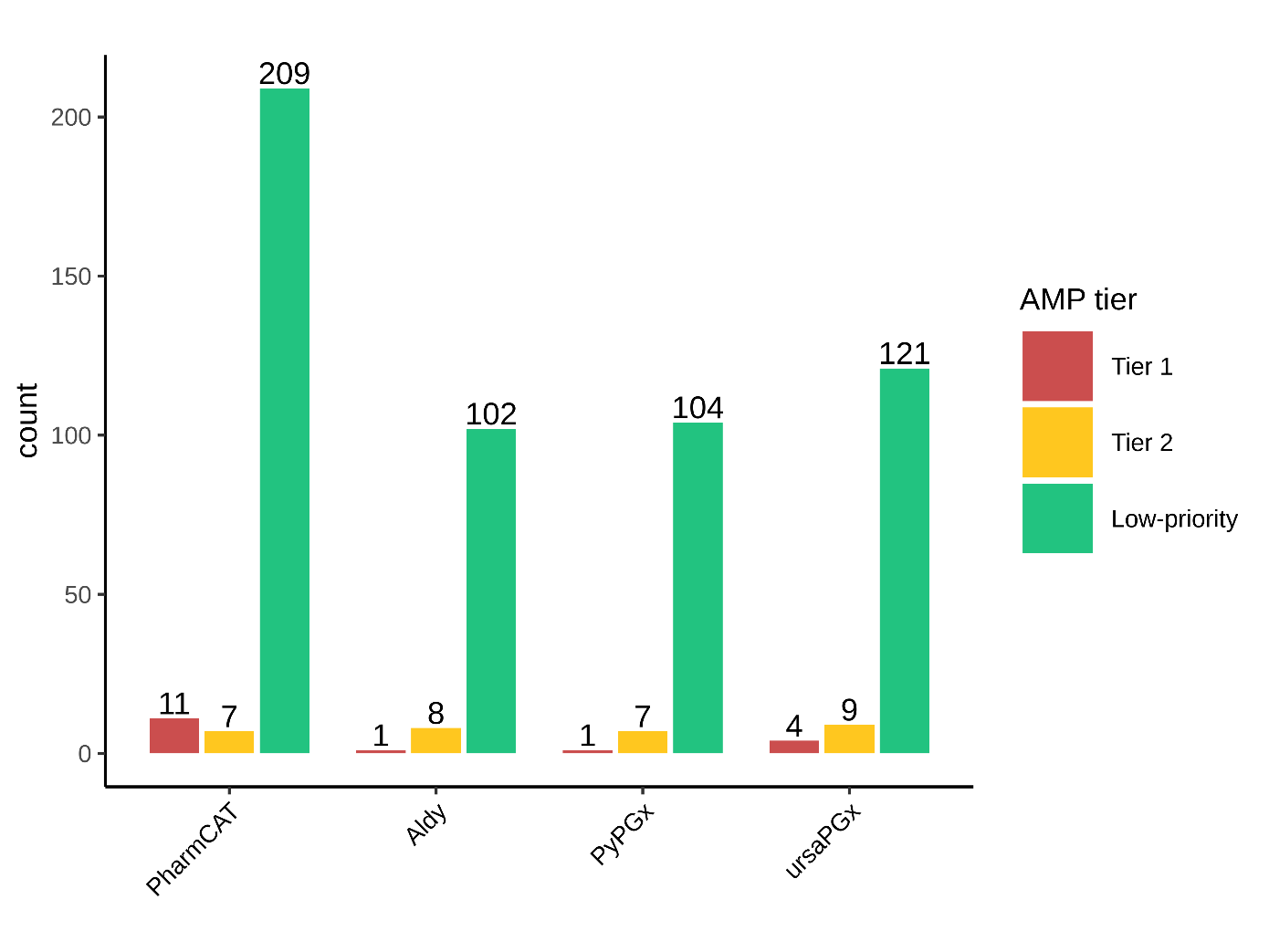


Barplot of the number of misidentified star-alleles per tool and per AMP tiers when using WGS data. Star alleles absent of tier 1 and tier 2 AMP sets were assigned to the Low-priority class.

**Figure S3.** Concordance of each star-allele caller with GeT-RM for diplotypes compared to DRAGEN.


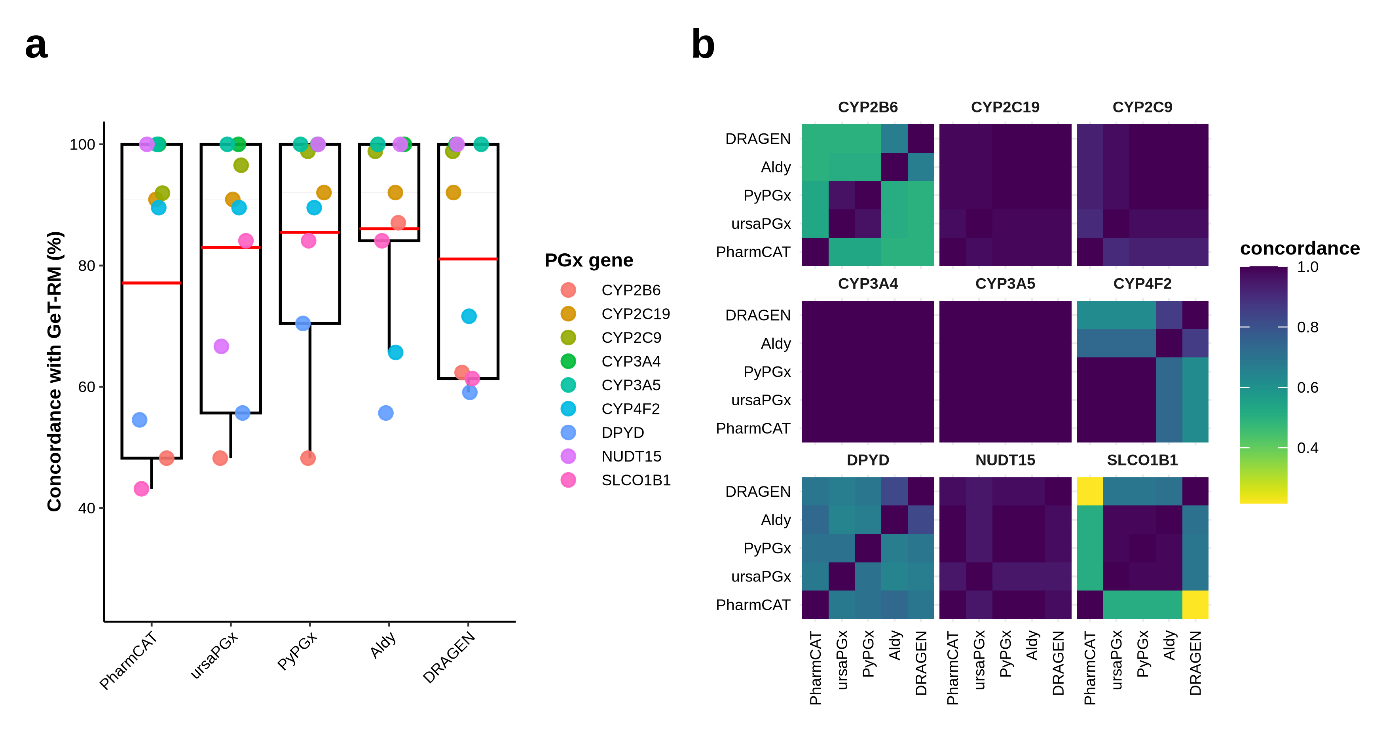


**a**: Diplotype concordance of each star-allele caller and DRAGEN with the GeT-RM consensus for each gene. The red line is the mean concordance. **c**: Pairwise diplotype concordance between each star-allele callers and DRAGEN for each gene.

**Figure S4.** Permutation test of the observed difference between concordance of African and European ancestry individuals.


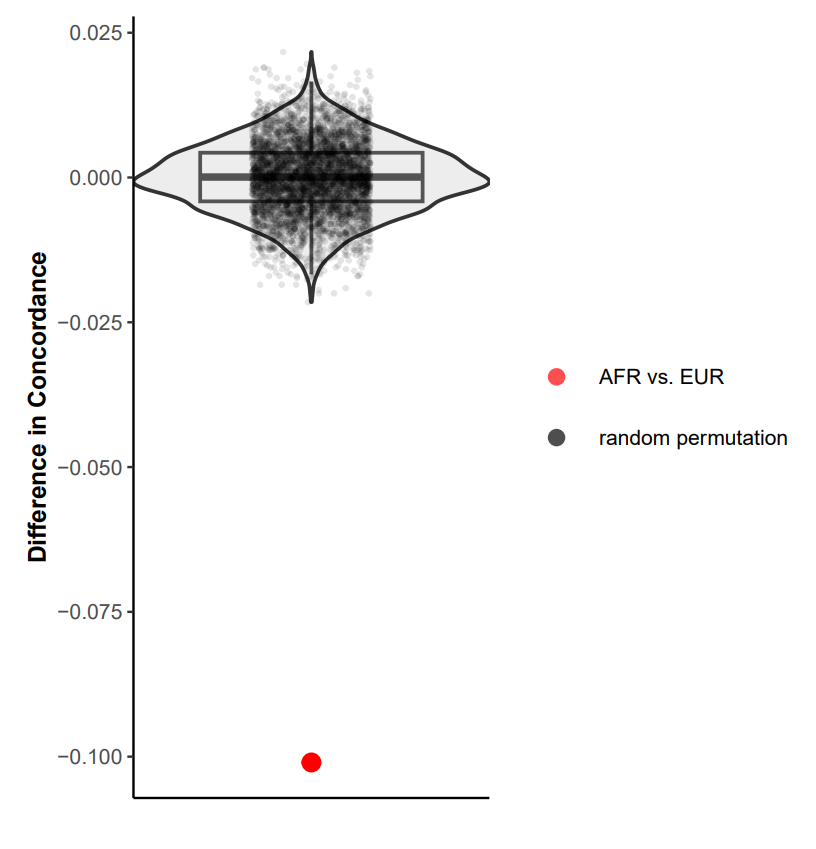


Permutation test to assess the significance of the observed difference between concordance for individuals of African ancestry (AFR) and concordance for individuals of European ancestry (EUR) between the four pipelines for the nine genes. To estimate the expect distribution under the null hypothesis, we randomly assigned to all AFR and EUR individuals the two population labels AFR and EUR, respecting the true group sizes, and computed the difference observed over n=5000 iterations of this random process. The red dot corresponds to the real difference observed.
